# Comparison of clinical characteristics of post-refractive surgery-related and post-herpetic neuropathic corneal pain

**DOI:** 10.1016/j.jtos.2020.07.006

**Published:** 2020-07-22

**Authors:** Betul N. Bayraktutar, M. Cuneyt Ozmen, Nasser Muzaaya, Gabriela Dieckmann, N. Dilruba Koseoglu, Rodrigo T. Müller, Andrea Cruzat, Bernardo M. Cavalcanti, Pedram Hamrah

**Affiliations:** a Center for Translational Ocular Immunology, Department of Ophthalmology, Tufts Medical Center, Tufts University School of Medicine, Boston, MA, USA; b Cornea Service, New England Eye Center, Department of Ophthalmology, Tufts Medical School Tufts University School of Medicine, Boston, MA, USA; c Ocular Surface Imaging Center, Cornea and Refractive Surgery Service, Massachusetts Eye and Ear Infirmary, Department of Ophthalmology, Harvard Medical School Boston, MA, USA

**Keywords:** Herpes keratitis, Neuropathic corneal pain, pain, Quality of life, Refractive surgery

## Abstract

**Purpose:**

To compare the clinical characteristics and in vivo confocal microscopy (IVCM) findings of patients with neuropathic corneal pain (NCP) due to refractive surgery (RS-NCP) and herpetic eye disease (H-NCP) to controls.

**Methods:**

Sixteen patients with RS-NCP and 7 patients with H-NCP, and 37 healthy reference age- and sex-matched healthy controls were included to the study. The medical records were reviewed for demographic features, detailed disease history, ocular surface disease index (OSDI), ocular pain assessment survey (OPAS) scores. IVCM images of patients were analyzed and compared to reference controls by two masked observers.

**Results:**

The mean pain intensity score for the last 24 h (5.1 ± 2.4 vs. 3.9 ± 1.2; p = 0.27), last 2 weeks (6.1 ± 2.5 vs. 4.8 ± 2.3; p = 0.13) for RS-NCP vs. H-NCP respectively, and quality of life scores (p = 0.23) were similar in both groups. Quality of life, especially mood (p = 0.06) and enjoying life/relations to others (p = 0.10) were affected in both groups, but were not statistically significant between groups. The mean total nerve density was lower in RS-NCP (5,702.4 ± 4,599.0 μm/mm^2^) compared to their respective controls (26,422.8 ± 4,491.0; p < 0.001) and in the H-NCP group (2,149.5 ± 2,985.9) compared to their respective controls (22,948.8 ± 3,169.0; p < 0.001). Alterations in DC density were similar between all groups (38.3 ± 48.0 cells/mm^2^ in RS-NCP, 61.0 ± 76.9 in H-NCP, p = 0.95).

**Conclusion:**

Neuropathic corneal pain patients due to refractive surgery show similar clinical characteristics, pain levels, quality of life impact, and IVCM findings as patients with NCP due to herpetic eye disease.

## 1. Introduction

Pain is defined as “unpleasant sensory and emotional experience associated with actual or potential tissue damage” and neuropathic pain (NP) is described as “pain caused by a lesion or disease of the somatosensory nervous system” by International Association for the Study of Pain [1]. Neuropathic corneal pain (NCP) is a new and ill-defined entity, which is characterized by dysfunctional corneal nerves causing non-specific symptoms, such as pain, burning, stinging, photophobia or severe dryness and failure of symptom resolution with conventional dry eye therapy [2–5]. The potential absence of objective slit-lamp findings or overlap with other ocular conditions makes NCP extremely difficult to diagnose.

Our knowledge about NCP, including underlying etiology, pathophysiological mechanism, severity of pain, its effect on quality of life (QoL), treatment and prognosis is very limited [2,6]. Alteration in ocular surface homeostasis, infections, or ocular surgery, among others, may lead to inflammation and peripheral nerve damage, resulting in hypersensitivity and peripheral sensitization (maladaptive nociceptor plasticity) [2–4,7,8]. If peripheral sensitization chronically continues, changes in the central nervous system occur, which lead to persistence of pain (central sensitization) as a result of changes in sensory, emotional, and other brain networks [2–4,7–9].

In addition to many systemic etiologies of NCP [2–5,10–12], underlying ocular etiologies associated with NCP may include dry eye disease (DED), infectious keratitis, herpes simplex keratitis, herpes zoster ophthalmicus, recurrent corneal erosion syndrome, radiation keratopathy, as well as trauma and ocular surgeries, such as cataract and refractive surgery procedures [2,3,7,13]. Refractive surgery is known as an effective method to correct refractive errors with a high predictability and is one of the most commonly performed surgical procedures in the United States [14,15]. However, dry eye symptoms as a consequence of refractive surgery are well described, and some patients may chronically suffer from these symptoms [16,17]. Furthermore, increased number of patients with unexplained ocular pain symptoms [2,3,7] are currently presenting or being identified after refractive surgery procedures [13]. Thus, post-refractive surgery neuralgia or NCP has become of interest to ophthalmologists and vision scientists alike [6,18,19]. While clinical and experimental studies on NCP are gradually increasing, the clinical characteristics, including pain levels and the impact on QoL need to be further elucidated [2–4,20,21]. Thus, we hypothesized that despite the varying etiologies, pain levels and quality of life impact of post-refractive surgery NCP are at least as severe as in post-herpetic neuralgia. Therefore, our aim in this study was to compare the clinical characteristics of NCP due to refractive surgery with the better-known post-herpetic neuralgia (NCP due to herpetic eye disease), in order to aid in understanding features of post-refractive surgery NCP. Elucidation of these aspects of this condition may assist ophthalmologists to better identify diagnosis and management needs, treatment targets and improved outcomes.

## 2. Methods

This is a cross-sectional, comparative, retrospective, case control study, with two comparison control groups, which was conducted at New England Eye Center, Department of Ophthalmology, Tufts Medical Center, Tufts University School of Medicine, Boston, MA. The study was approved by Institutional Review Board/Ethics Committee of Tufts Medical Center/Tufts University Health Sciences and the study protocol conformed to the Declaration of Helsinki, and adhered to the Health Insurance Portability and Accountability Act (HIPAA).

### 2.1. Patients

The medical records and in vivo confocal microscopy (IVCM) images of patients who were diagnosed as NCP by the same experienced clinician (PH) between January 2015 and April 2019 were evaluated retrospectively. Diagnosis of NCP was made based on presence of neuropathic ocular symptoms (burning, stinging, photophobia, pain, severe dryness), absent or minimal slit-lamp findings to explain symptoms, corneal nerve abnormalities as detected by IVCM (HRT3/RCM, Heidelberg Engineering GmbH, Heidelberg, Germany). Subjects with refractive surgery related NCP (RS-NCP) and herpetic eye disease-related NCP (H-NCP) were included in the analysis. Patients were excluded if they had any other ocular pathology that might have resulted in pain, such as active corneal infections, abrasions, angle-closure glaucoma, and anterior uveitis, or if they had NCP with a different etiology rather than refractive surgery or herpetic eye disease. Age- and sex-matched reference controls in this study were healthy, asymptomatic individuals, with no ocular pathology, absent ocular surface staining, and tear film break-up time of more than 10 s. All controls were drawn from an IRB-approved prospective normative study database that enrolled healthy subjects after having a complete history and ocular examination. Matching our control group to the other 2 groups in terms of age and sex resulted in the 37 participants from this database. Reference controls were classified as controls for the refractive surgery group (C-1) if their age was ≥ 20 and ≤ 50 and controls for the herpetic group (C-2) if they were > 50 years of age. This subgroup classification was needed for assessment of IVCM images, as the mean age of the population for the refractive surgery patients with NCP was different and less than NCP patients with a history of herpetic eye disease and due to the fact that nerve density may decrease with increasing age.

### 2.2. Clinical chart review

Demographic features of the patients, time of insult, time between insult and pain onset, duration of pain (time between first consistent pain experience described by patient and the first visit date at our center. Duration of non-specific ocular symptoms and inconsistent pain was not included), Ocular Surface Disease Index (OSDI) [22], Ocular Pain Assessment Survey (OPAS) scores [23], proparacaine challenge test (PCT) results at the initial visits were recorded. For the PCT [2,3], patients were asked to report their pain relief based on visual analogue scale after 1 min of installation of 0.5% proparacaine hydrochloride eye drops (Alcaine; Novartis Ophthalmics, East Hanover, NJ). The PCT was performed in the presence of pain at the day of the visit and results were reported from the initial visit in our clinic. Given that this test had not been routine prior to 2017, not all patients had received the test. Based on complete relief, partial relief, or no relief in symptoms after the PCT, patients were grouped as peripheral, mixed and central NCP, respectively [2,3]. This classification was used in the patients who had PCT at the initial visit, but not in patients for which the PCT was not available.

### 2.3. In vivo confocal microscopy and image analysis

Laser IVCM images were conducted on central corneas of all patients and controls, bilaterally, as previously described [24]. Equipped with a 63 × objective immersion lens with a numerical aperture of 0.9 (Olympus, Tokyo, Japan), this microscope uses a 670-nm red wavelength diode laser source to produce an image representing a coronal section of the cornea of 400 × 400 μm (horizontal x vertical). Digital images are recorded at of 30 frames/s. Adjacent images are separated by 1 μm, with a lateral resolution of 1 μm/pixel. To perform this procedure, both eyes were topically anesthetized using 0.5% proparacaine hydrochloride (Alcaine; Novartis Ophthalmics). This was followed by administration of a drop of hydroxypropyl methylcellulose 2.5% (GenTeal gel, Alcon, Fort Worth, TX) to improve the optical coupling with the cornea module of the microscope. The cornea module was mounted with a disposable, sterile polymethylmethacrylate cap (Tomo-Cap; Heidelberg Engineering GmbH), filled with a layer of hydroxypropyl methylcellulose 2.5% (GenTeal gel; Alcon), gel was also applied to the surface of the cap. The equipment is manually advanced until the gel on the cap comes in contact with the surface of the central cornea.

Out of a total of six to eight sequence scans performed on the full thickness of the central cornea, resulting in a total of 50–100 images of the corneal subbasal layer, a masked observer (B.N.B.) selected the three most representative images (best focused, single layer, minimum folds and good contrast) of the subbasal nerve plexus. Two masked observers (B.N.B.; N.M.) analyzed IVCM images for morphology and density of dendritiform cells (DCs) and subbasal nerve plexus (SNP). In case of any discrepancy, the images were analyzed and adjudicated by a third observer. The DC density was measured using Image J (https://imagej.nih.gov/ij/) as previously described [24], and total, trunk, branch nerve density were measured using Neuron J (a semi-automated tracing plugin for Image J) [24]. The IVCM image analyses results were then compared to age- and sex-matched healthy reference controls.

### 2.4. Questionnaires

The OSDI is a validated 12-item questionnaire with 3 dimensions for rapid assessment of the ocular irritation related to dry eye disease and its impact on vision related functioning [22]. Each question is graded between 0 and 4 (0 = none of the time, 1 = some of the time, 2 = half of the time, 3 = most of the time, 4 = all of the time). The overall OSDI is scored on a scale of 0–100 in which higher scores show more disability.

The OPAS is a validated multidimensional questionnaire for ocular pain [23], which includes 6 dimensions, including eye pain intensity for the last 24 h, eye pain intensity for the last 2 weeks, non-eye pain intensity, quality of life, aggravating factors, associated factors and symptom relief. Patients were asked to respond all questions according pain scale between 0 and 10 (0 = no pain, 10 = worst pain ever) [23].

### 2.5. Statistical analyses

Statistical analyses were performed with SPSS software version 22.0 (SPSS Inc., IBM, Chicago, IL, USA). Distribution of data was analyzed by Kolmogorov-Smirnov test. Mann Whitney *U* test and Chi-squared were used to assess the differences in demographic and clinical parameters of RS-NCP and H-NCP groups. The differences in IVCM parameters in patients and healthy groups were compared by Kruskal Wallis-test with pairwise comparison test if needed. Confounding factors (age and sex) were controlled by using a generalized linear model. P values lower than 0.05 were considered statistically significant.

## 3. Results

Sixty patients were included in the study. Sixteen patients (7 females and 9 males) with post-refractive surgery neuropathic corneal pain (RS-NCP), 7 patients (4 females and 3 males) with post-herpetic neuropathic corneal pain (H-NCP), and thirty-seven healthy (15 young controls; 4 females and 11 males and 22 old controls; 10 females and 12 males) controls were enrolled to the study. The mean age of the patients was 39.7 ± 13.4 years (25.0–66.0 years), 70.7 ± 12.8 (56.0–89.0 years), 33.6 ± 9.5 (22.0–49.0 years) and 61.0 ± 6.8 (51.0–74.0 years) in RS-NCP, H-NCP, C-1 and C-2 controls, respectively (p = 0.61 for RS-NCP vs. C-1, p = 0.21 for H-NCP vs. C-2, and p = 0.001 for RS-NCP vs. H-NCP). Demographic features of patients are summarized in Table 1.

### 3.1. Clinical characteristics

In the RS-NCP group, 1 patient had laser epithelial keratomileusis (LASER), 2 patients had photorefractive keratectomy (PRK) and 13 patients had laser assisted in-situ keratomileusis (LASIK) surgery prior to NCP. In the H-NCP group, 5 patients developed NCP after herpes zoster ophthalmicus and 2 patients developed NCP after herpes simplex keratitis.

A history of DED prior to surgery was recorded in 3 (18.7%) of RS-NCP patients (2 of them developed DED after surgery and 1 had surgery with history of DED) and in 2 (28.5%) of H-NCP patients. In the RS-NCP group, there was no predominance for any systemic condition, but depression in 2 patients (12.5%), trigeminal neuralgia in 1 patient (6.2%), chronic inflammatory demyelinating polyneuropathy in 1 patient (6.2%), and small fiber neuropathy in 1 patient (6.2%) were recorded. The most common systemic disorder recorded in H-NCP group was hyperlipidemia (4 patients, 57.1%) and in 1 patient (14.2%) depression was recorded. Detailed demographic and clinical features of patients were shown in Tables 2 and 3.

The mean time between insult and pain was 9.4 ± 17.3 weeks (range: 0.1–68.0 weeks) in the RS-NCP and 45.0 ± 32.5 weeks (range: 2.0–96.0 weeks) in H-NCP group (p = 0.006). The mean duration of pain at the visit was 89.3 ± 75.3 weeks (range: 1.0–252.0 weeks) in RS-NCP and 172.0 ± 183.3 weeks (range: 6.0–416.0 weeks) in H-NCP and (p = 0.59) (Tables 1–3).

Proparacaine challenge test results were recorded in 10 RS-NCP patients (55.5%) and in 3 H-NCP patients (42.8%). In the RS-NCP group, 4 patients (40.0%) had peripheral NCP, 4 patients (40.0%) had mixed NCP and 2 patients (20.0%) had central NCP, whereas, in the H-NCP group, 2 patients had mixed NCP (66.6%) and 1 patient had central NCP (33.3%) (p = 0.42).

### 3.2. Corneal nerve alterations by in vivo corneal confocal microscopy

**Fig. 1.**
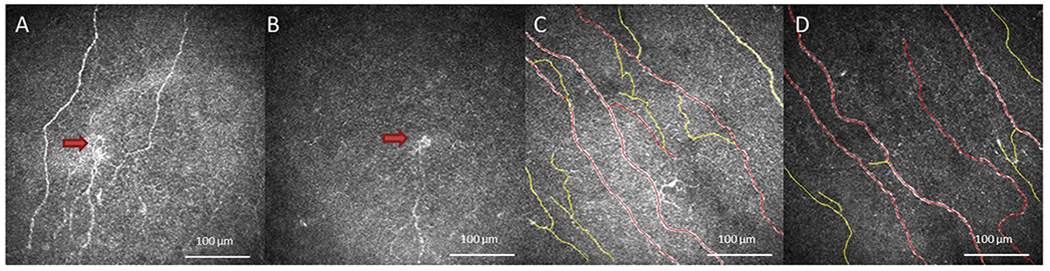
Laser in Vivo Confocal Microscopy (IVCM) Images of Corneal Nerves in Patients and Controls. IVCM images obtained at the level of corneal subepithelial nerve plexus demonstrate changes in corneal nerves in patients and healthy controls. Decreased corneal nerve density, decreased corneal nerve branching, and presence of microneuroma (red arrow) were observed in RS-NCP (A). Decreased corneal nerve density and microneuroma (red arrow) were observed in H-NCP patients (B). Subbasal nerves traced by Neuron J in healthy reference control groups C-1 (C) and C-2 (D) (red tracing; trunk nerve fibers, yellow tracing; branch nerve fibers).

The mean total, trunk and branch subepithelial nerve plexus density are shown in Table 4 and Fig. 1. The mean total nerve density was 5,702.4 ± 4,599.0 μm/mm^2^ (range: 234.2–15,257.8) and 2,149.5 ± 2,985.9 (range: 0.0–7,866.5) in RS-NCP and H-NCP respectively, compared to 26,422.8 ± 4,491.0 (range: 37,063.9) in C-1 and 22,948.8 ± 3,169.0 (range: 18,228.4–28,991.9), in C-2 groups.

**Fig. 2.**
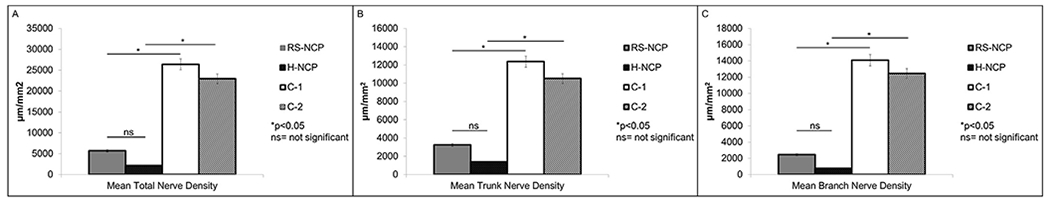
Comparison of total (A), trunk (B) and branch (C) nerve densities detected by in vivo confocal microscopy in RS-NCP, H-NCP and healthy reference controls.

The mean total (p < 0.001), trunk (p < 0.001) and branch (p < 0.001) nerve densities were lower in RS-NCP compared to C-1. The mean total (p < 0.001), trunk (p < 0.001) and branch (p < 0.001) nerve densities were lower in H-NCP compared to C-2. However, total (p = 0.30), trunk (p = 0.32) and branch (p = 0.97) nerve densities were similar in RS-NCP and H-NCP (Figs. 1 and 2). The mean total (p = 0.75), trunk (p = 0.66) and branch (p = 0.43) nerve density were not different in age based control groups.

### 3.3. Dendritiform cell density by in vivo corneal confocal microscopy

**Fig. 3.**
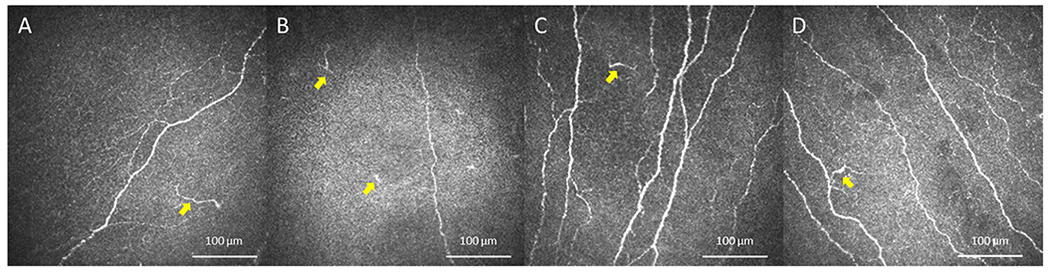
In Vivo Confocal Microscopy (IVCM) images of Dendritiform Cells in Patients and Controls. IVCM images of dendritiform cells (yellow arrows) showed no difference between patients with RS-NCP (A), H-NCP (B) and healthy reference control groups C-1 (C) and C-2 (D).

**Fig. 4.**
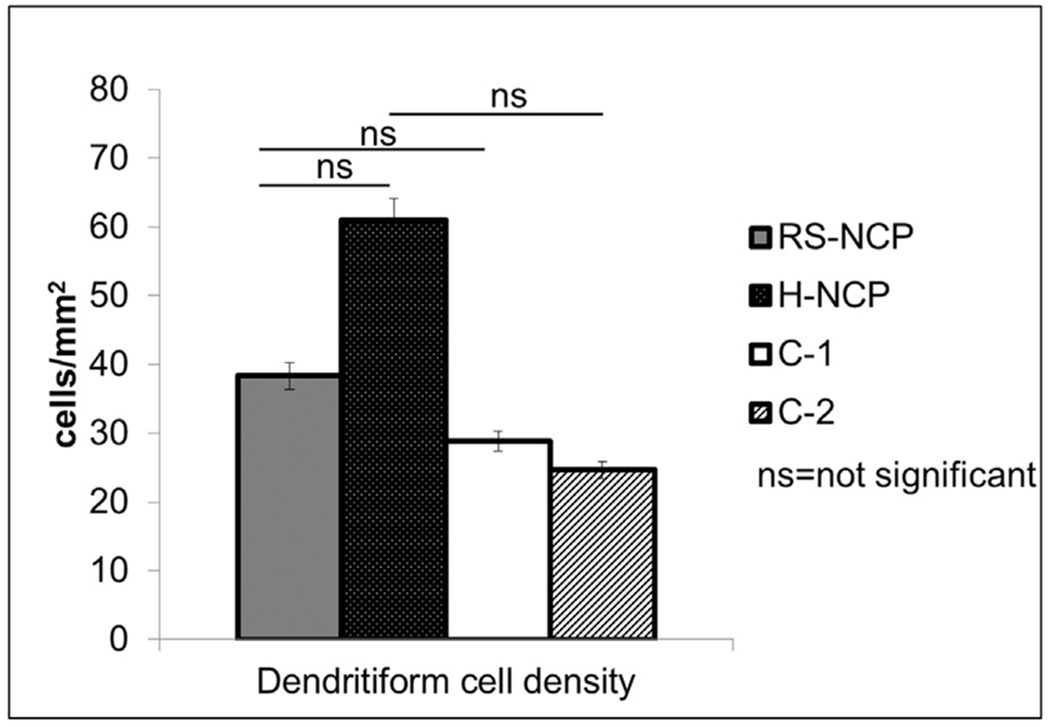
Comparison of dendritiform cell densities detected by in vivo confocal microscopy in RS-NCP, H-NCP and healthy reference controls.

The mean DCs densities are shown in Table 4. No statistically significant difference was found between groups regarding DC density (p = 0.95) (Figs. 3 and 4).

### 3.4. Questionnaires

The mean OSDI score and the mean pain intensity scores calculated from OPAS are presented in Table 5. The mean OSDI score (p = 0.10), the mean pain intensity score for the past 24 h (p = 0.27) (when it was most; p = 0.24 or least painful; p = 0.37), the mean pain intensity score in the past 2 weeks (p = 0.13) (when it was most; p = 0.08 or least painful; p = 0.57), and non-eye pain in the past 24 h (p = 0.79) and in the past 2 weeks (p = 0.62) were not statistically different between RS-NCP and H-NCP (Tables 5 and 6).

The mean effect of pain on reading and/or computer use (p = 0.53), driving and/or watching TV (p = 0.63), general activity (walking, doing house chores) (p = 0.78), mood (p = 0.06), sleep (p = 0.18), enjoying life/relations with other people (p = 0.10), and time spent thinking about eye pain (p = 0.83) did not show statistical difference between RS-NCP and H-NCP patients (Table 6). However, mood, sleep, and enjoying life were more affected by pain in the RS-NCP group compared to the H-NCP group (Table 6).

In both groups, patients reported that they spent most of their time by thinking about their eye pain (72.3% in RS-NCP and 77.1% in H-NCP, p = 0.83). Aggravating factors such as wind, dry air, heat and air conditioning (p=0.40) and volatile chemicals (p=0.75) had a similar effect on pain in both groups (Table 6). Redness (p = 0.29), burning (p = 0.10), sensitivity to light (p = 0.36) and tearing (p = 0.73) accompanied to pain with comparable frequency in RS-NCP and H-NCP (Table 6). Burning was the most associated symptom with pain in both groups (54.3% in RS-NCP and 80.0% in H-NCP, p = 0.10). The mean quality of life score (QoL, questions 13–19) (p = 0.23), aggravating factors (questions 20–21) (p = 0.58) and associated factors (questions 22–25) (p = 0.36) scores calculated from OPAS were all affected in both groups and did show similar findings (Table 5).

## 4. Discussion

All NP disorders have a common denominator (damage or malfunctioning of somatosensory nervous system) with different underlying etiologies and pathogenesis [25,26]. Pain perception begins with detection of noxious stimuli by nociceptors and induction of action potentials created by nociceptor to somatosensory cortex and paralimbic structures [2]. Peripheral axonal injury may result in release of pro-inflammatory mediators, which may lower the threshold of action potentials of nociceptors, spread the stimuli to adjacent nociceptor and activate silent nociceptor [2,6,12]. Increased peripheral sensitization leads to central sensitization over time, which may result in increased pain levels and awareness [2,6,12]. Trauma, inflammation and iatrogenic damage may trigger this pain signaling pathway [4,6,27].

It is well-known that herpes simplex [28], herpes zoster [29] and refractive surgery [30] can cause corneal nerve damage, which can be detected as decreased nerve density by IVCM. Post-herpetic neuralgia stems from damage to peripheral and central neurons that may be due to either inflammation and/or viral infection, and it is known as one of the most painful conditions [31–35]. Although the classical presentation of post-herpetic neuralgia is pain starting with acute viral disease (acute) and persisting chronically, subacute and chronic neuralgia may also occur, and occasionally pain may develop after a pain-free period [36–38] as presented in our H-NCP group. Moreover, chronic post-herpetic neuralgia does not develop with all acute episodes and can occur after several acute episodes. Interestingly, even long-term follow-up has shown that nerve density may not be restored to healthy levels after herpetic eye disease [28,29]. Similarly, persistent nerve loss over many years has been shown following refractive surgery [17,30,39–41]. Furthermore, stimulated keratocytes during refractive surgeries may produce several cytokines, chemokines, and growth factors that might initiate corneal inflammation after surgical procedures [42,43]. Corneal nerve damage and post-operative inflammation are most likely involved in the development of NCP after refractive surgery, similar to post-herpetic neuralgia.

Herein, we present and compare clinical characteristics of post-refractive surgery NCP and post-herpetic neuralgia patients. Both refractive surgery and herpetic eye disease related NCP present with moderate to severe pain levels, which affect quality of life negatively and significantly. OSDI scores showed that RS-NCP and H-NCP patients suffered from ocular discomfort symptoms with a similar frequency. Further, pain intensity scores showed that pain following refractive surgery was at least as severe as H-NCP patients. Pain levels of patients were strong enough to have moderate impact on daily activities such as reading, computer use, driving, watching TV, walking, and doing house chores. The pain not only affects daily activities, but also mood, enjoying life, and relations with other people, particularly in the post-refractive surgery group.

Although, QoL scores and the sub-questions (reading and/or computer use, driving and/or watching TV, general activities like walking, doing house chores, mood, sleep, enjoying life/relations with other people) are similar in both groups, RS-NCP patients were affected more significantly. However, in contrast to QoL, RS-NCP patients were affected less by aggravating factors (wind, dry air, heat, air conditioning, and volatile chemicals). Furthermore, RS-NCP patients’ symptoms were less likely to be associated with other ocular surface symptoms such as redness, burning, sensitivity to light and tearing compared to H-NCP. When comparisons have been made between other serious chronic conditions including cancer, cardiovascular or neuromuscular disorders and chronic pain, it has been showed that chronic pain conditions had at least as much impact on QoL as those conditions [44,45]. NCP symptoms are generally much more severe and persistent than dry eye symptoms [46–48], which as another chronic ocular condition has shown utility scores similar to moderate angina [49].

Previous studies have shown that negative mood also might influence the experience of neuropathic pain [50,51]. Diaries from patients with reflex sympathetic dystrophy (complex regional pain syndrome) demonstrated that yesterday’s depressed mood contributed today’s increased pain and yesterday’s pain also contributed today’s depression, anxiety and anger [51]. Moreover, it has been shown that better baseline emotional health and physical functioning are likely to respond to treatment better in painful neuropathies [52–54]. The same vicious cycle may be present in NCP patients, which makes the treatment more challenging and also more critical, as NCP patients may be at risk to develop mood disorders.

In our NCP patients, peripheral nerve damage was detected in both groups by IVCM. The nerve densities were comparable in RS-NCP and H-NCP patients. However, both groups demonstrated significantly lower nerve densities than their respective age- and sex-matched healthy controls groups. Previous studies have shown that although corneal nerve regeneration occurs in patients with herpetic eye disease, it still remains low as compared to age-matched healthy individuals, even after 3 years of follow-up [28]. Further, it has been reported that reaching normal corneal nerve density levels can be achieved after 2 years of PRK and by 5 years following LASIK surgery [55]. The results warrant future studies to compare corneal nerve alterations between post-herpetic patients with and without NCP, as well as in post-refractive surgery patients with and without NCP to assess if corneal nerve alterations are specific to NCP or the underlying condition. Although, exact mechanisms underlying corneal nerve regeneration are not completely understood, neuropeptides, neurotrophins, and growth factors released by corneal epithelium and keratocytes, including nerve growth factor (NGF), brain derived neurotrophic factor (BDNF), ciliary neurotrophic factor (CNTF), neurotrophin 3, neurotrophin 4/5, epidermal growth factor (EGF), and glial cell derived neurotrophic factor (GDNF) seem to have role in nerve fiber survival, differentiation and maturation [56,57]. However, delayed or abnormal corneal nerve regeneration after refractive surgery or herpetic eye disease may precipitate development of NCP. Furthermore, corneal inflammation induced by surgical trauma during refractive surgery [13,58] and during the course of herpetic infection [59] contribute to disease processes. Interestingly, in our patient population, DC alterations as shown by IVCM did not show a significant difference compared to healthy individuals. Notably, the presentation times between refractive surgical procedure/active herpetic disease and the initial study visit were widely distributed, which may have influenced the level of corneal inflammation. However, inflammation is required for sensitization of peripheral nerves and the development of NCP, but is not necessarily required to be maintained during the course of the disease after peripheral and central sensitization of neuronal pathways have been initiated. Accordingly, DC densities in some of our NCP patients that were on topical steroids and cyclosporine A during the study visits, may have confounded by topical therapies without resolution of pain.

The goal of NCP treatment should be to reduce pain in the short term and to inhibit and/or resolve central sensitization in the long term. Therefore, treatment of NCP requires differentiation of peripheral and central component pain for adequate management [2,3,12]. In peripheral NCP patients, topical ocular surface treatments, such as artificial tears, topical steroids [3], autologous serum tears [60,61], cryopreserved amniotic membrane [62], and bandage contact lenses may be sufficient by down-regulating ectopic, spontaneous, or hypersensitive signaling in peripheral nociceptor pathways [13]. However, central sensitization requires systemic pharmacotherapies, such as tricyclic antidepressants, low dose-naltrexone, anticonvulsants, serotonin-norepinephrine inhibitors, and other analgesics addition to topical ocular surface treatments [3,63]. The proparacaine challenge test is used to assess central component of pain as previously described [3]. Despite our limited data for proparacaine challenge test, available patient results show that the majority of RS-NCP patients had at least a nonocular component of pain that did not resolve with anesthetic drops. Similar to our patients, previous studies have also reported different amount of centralization component in refractive surgery related NCP, which suggests severe pain, longer and complex treatment and more diminished quality of life [9,12,13,64,65]. A high rate of centralization suggest delayed diagnosis and treatment in both groups. Thus, in order to prevent central sensitization and impairment of quality of life and mood, early diagnosis and treatment have a crucial importance in NCP, especially in a younger age group with a more active work and social life like refractive surgery patients.

In conclusion, despite the limitations of our study, including the retrospective design, relatively small sample size, and heterogeneity of the groups, our study suggests that RS-NCP patients may present with a wide spectrum of clinical features similar to post-herpetic neuralgia. However, pain severity, impaired quality of life and mood, severe nerve damage and the presence of central sensitization are dominant findings in these patients. Therefore, NCP should be kept in mind in refractive surgery patients with non-specific persistent symptoms, who are unresponsive to conventional dry eye treatments and additional evaluation, such as IVCM to assess nerve abnormalities, and the proparacaine challenge test to assess central component of symptoms, should be considered for diagnosis and management purposes. Early diagnosis prior to the development of central sensitization is thus of great importance.

## Figures and Tables

**Table 1:** Demographic features of patients and control groups.

Parameter	RS-NCP	H-NCP	C-1	C-2	P value
N	16	7	15	22	
Age (years)	39.7 ± 13.4 (25.0–66.0)	70.7 ± 12.8 (56.0–89.0)	33.6 ± 9.5 (22.0–49.0)	61.0 ± 6.8 (51.0–74.0)	RS-NCP vs. C-1, p = 0.61; H-NCP vs. C-2, p = 0.21; RS-NCP vs. H-NCP, p = 0.001
Gender (female/male)	7/9	4/3	4/11	10/12	0.21
Time between insult and pain onset (weeks)	9.4 ± 17.3 (0.1–68.0)	45.0 ± 32.5 (2.0–96.0)	–	–	0.006
Duration of pain (weeks)	89.3 ± 75.3 (1.0–252.0)	172.0 ± 183.3 (6.0–416.0)	–	–	0.59

RS-NCP: Neuropathic corneal pain due to refractive surgery; H-NCP: Neuropathic corneal pain due to herpetic eye disease; C-1: Control group 1; C-2: Control group 2.

**Table 2:** Clinical characteristics of post-refractive surgery neuropathic corneal pain patients.

Patient No	Age	Gender (female/male)	Ethnicity	Lateralization (right/left)	Insult	Complication Related to Insult	Time between insult and pain onset (weeks)	Duration of pain (weeks)	Ocular Surgery (except insult)	Accompanying Ocular Disease	Accompanying Systemic Disease	Proparacaine Challenge Test (% relief)	Topical Treatment
1	25	F	Caucasian	R	LASEK	NA	8	44	NA	NA	NA	0%	AST
2	25	M	Caucasian	R	PRK	NA	12	172	Strabismus Ptosis	Oculocutaneous Albinism	Oculocutaneous Albinism	100%	NA
3	27	M	Caucasian	R	PRK	Corneal abrasion	1	4	NA	NA	NA	NA	PFAT
4	48	M	Caucasian	L	LASIK	NA	Immediately	252	NA	NA	NA	NA	AST, PFAT
5	27	M	Hispanic	L	LASIK	NA	Immediately	124	NA	NA	Depression	NA	CsA
6	60	F	Caucasian	L	LASIK	NA	Immediately	104	NA	NA	Trigeminal Neuralgia	33.3%	CsA, PFAT
7	33	M	Caucasian	R	LASIK	NA	12	11	NA	NA	Chronic Inflammatory Demyelinating Polyneuropathy	75.0%	AST, PFAT, 0.5% loteprednol etabonate
8	66	M	Caucasian	L	LASIK	NA	1	36	Cataract Extraction	Fuchs Endothelial Dystrophy	NA	100%	AST
9	33	F	Caucasian	L	LASIK	NA	1	64	NA	NA	Depression	NA	AST, 0.5% loteprednol etabonate
10	29	M	Caucasian	R	LASIK	NA	Immediately	72	NA	NA	NA	0%	AST,PFAT
11	45	F	Caucasian	R	LASIK	Flap stria	26	1	NA	NA	Reynaud phenomenon	100%	PFAT, 1% prednisolone acetate
12	60	F	Caucasian	L	LASIK	NA	Immediately	96	Blepharoplasty	DED	Small fiber neuropathy, Hypothryroidism, Postural Orthostatic Tachycardia, Gastroesophageal Reflux	50%	PFAT
13	42	M	Asian	R	LASIK	Epithelial ingrowth removal, DED	16	224	NA	DED	NA	NA	PFAT
14	36	F	Caucasian	R	LASIK	NA	Immediately	36	NA	NA	Thyroid Disorder	10.0%	NA
15	32	F	Caucasian	R	LASIK	Flap stria	6	128	NA	NA	NA	100%	AST, PFAT
16	48	M	Caucasian	R	LASIK	Flap dislocation (replacement performed), DED	68	61	NA	DED	Hyperlipidemia	NA	AST, 0.5% loteprednol etabonate

AST: Autologous serum tears, CsA: 0.05% cyclosporine A, DED: Dry eye disease, LASEK: Laser epithelial keratomileusis, LASIK: Laser assisted in situ keratomileusis, NA: Not applicable, PFAT: Preservative free artificial tears.

**Table 3:** Clinical characteristics of post-herpetic neuropathic corneal pain patients.

No	Age	Gender (female/male)	Ethnicity	Lateralization (right/left)	Insult	Time between insult and pain onset (weeks)	Duration of pain (weeks)	Ocular Surgery	Accompanying Ocular Disease	Accompanying Systemic Disease	Proparacaine Challenge Test (% relief)	Topical Treatment
1	65	M	Caucasian	L	Herpes zoster	23	20	NA	NA	NA	NA	PFAT, 0.5% loteprednol etabonate
2	56	F	Caucasian	L	Herpes Zoster	40	6	NA	NA	Depression Anxiety and Crohn’s Disease	NA	AST, PFAT, 0.5% loteprednol etabonate
3	88	M	Caucasian	R	Herpes Zoster	63	NA	Cataract Extraction	NA	Osteoarthritis Hyperlipidemia Hypertension Gastroesophageal Reflux	NA	AST, 1% prednisolone acetate
4	66	M	Caucasian	L	Herpes Zoster	2	158	Cataract Extraction and Pars Plana Vitrectomy, Ahmed Glaucoma Valve Implantation	DED Glaucoma (steroid responder)	NA	95.0%	AST, PFAT, 0.5% loteprednol etabonate
5	62	F	Hispanic	R	Herpes Simplex	NA	380	Penetrating Keratoplasty, Ahmed Glaucoma Valve	Glaucoma (steroid responder)	Hyperlipidemia	22.2%	0.5% loteprednol etabonate
6	69	F	Caucasian	L	Herpes Simplex	96	416	Cataract Extraction	DED LSCD	Scleroderma Sjögren’s Syndrome Hyperlipidemia	NA	PFAT
7	89	M	Caucasian	L	Herpes Zoster	46	52	Cataract Extraction	NA	Hyperlipidemia Hypertension Chronic renal failure	0%	AST, 0.5% loteprednol etabonate

AST: Autologous serum tears, DED: Dry eye disease, LSCD: Limbal stem cell deficiency, NA: Not applicable, PFAT: Preservative free artificial tears.

**Table 4:** In vivo confocal microscopy parameters in post-herpetic neuropathic corneal pain, post-refractive neuropathic corneal pain and control groups.

IVCM parameters	RS-NCP (n = 16)	H-NCP (n = 7)	C-1(n = 15)	C-2 (n = 22)	p value	pairwise comparison p values
Total nerve density (μm/mm^2^)	5,702.4 ± 4,599.0 (234.2–15,275.8)	2,149.5 ± 2,985.9 (0.0–7,866.5)	26,422.8 ± 4,491.0 (21,565.0–37,063.9)	22,948.8 ± 3,169.0 (18,228.4–28,991.9)	< 0.001	RS-NCP vs. C-1, p < 0.001; H-NCP vs. C-2, p < 0.001; RS-NCP vs. H-NCP, p = 0.30
Trunk nerve density (μm/mm^2^)	3,243.1 ± 2,372.1 (0.0–6,781.6	1,399.6 ± 1,847.6 (0.0–5,007.5	12,353.2 ± 2,516.5 (8,024.4–17,772.1)	10,511.7 ± 1,468.3 (8,664.4–15,382.5)	< 0.001	RS-NCP vs. C-1, p < 0.001; H-NCP vs. C-2, p < 0.001; RS-NCP vs. H-NCP, p = 0.32
Branch nerve density (μm/mm^2^)	2,459.2 ± 2,360.8 (0.0–8,476.2)	749.8 ± 1,159.8 (0.0–2,858.9)	14,069.6 ± 3,414.2 (8,149.7–19,291.7)	12,437.0 ± 2,618.9 (8,632.9–18,417.1)	< 0.001	RS-NCP vs. C-1, p < 0.001; H-NCP vs. C-2, p < 0.001; RS-NCP vs. H-NCP, p = 0.97
DC density (cell/mm^2^)	38.3 ± 48.0 (0.0–141.6)	61.0 ± 76.9 (2.0–189.5	28.9 ± 29.8 (2.0–95.8)	24.6 ± 19.9 (2.0–70.8)	0.95	–

RS-NCP: Neuropathic corneal pain due to refractive surgery, H-NCP: Neuropathic corneal pain due to herpetic eye disease, C-1: Control group 1, C-2: Control group 2.

**Table 5:** Symptom questionnaire results for post-herpetic and post-refractive surgery neuropathic corneal pain patients.

	RS-NCP (n = 16)	H-NCP Group (n = 7)	p value
**OSDI**			
Mean ± SD (range)	59.1 ± 19.8 (14.5–90.9)	60.0 ± 23.8 (22.9–100.0)	0.10
**Pain Intensity for the last 24 h**			
Mean ± SD (range)	5.1 ± 2.4 (1.0–9.6)	3.9 ± 1.2 (2.0–5.6)	0.27
**Pain Intensity for the last 2 weeks**			
Mean ± SD (range)	6.1 ± 2.5 (1.6–9.6)	4.8 ± 2.2 (2.0–9.0)	0.13
**QoL score**			
Mean ± SD (range)	6.1 ± 2.4 (2.0–9.5)	4.7 ± 2.5 (1.8–9.0)	0.23
**Aggravating Factors**			
Mean ± SD (range)	4.3 ± 3.2 (0.0–10.0)	5.5 ± 3.4 (2.5–10.0)	0.58
**Associated Factors**			
Mean ± SD (range)	3.8 ± 2.5 (0.0–10.0)	4.4 ± 1.8 (2.0–7.0)	0.36

RS-NCP: Neuropathic corneal pain due to refractive surgery, H-NCP: Neuropathic corneal pain due to herpetic eye disease, OSDI: Ocular surface disease index, QoL: Quality of life, SD: standard deviation.

**Table 6:** Question by question analysis of Ocular Pain Assessment Survey.

Ocular Pain Assessment Survey Questions (0–10) (0 = no pain, 10 = worst pain)	RS-NCP (n = 16)	H-NCP Group (n = 7)	p value
**Q1 - The overall severity of your pain today**			
Mean ± SD (range)	5.4 ± 3.3 (0.0–10.0)	5.1 ± 2.8 (2.0–10.0)	0.76
**Q4 - Level of pain when it is most painful in the past 24 h**			
Mean ± SD (range)	6.7 ± 2.3 (2.0–10.0)	5.5 ± 2.5 (2.0–10.0)	0.24
**Q5 - Level of pain when it is least painful in the past 24 h**			
Mean ± SD (range)	3.6 ± 2.9 (0.0–9.0)	2.2 ± 1.2 (1.0–4.0)	0.37
**Q6 - Level of pain on average in the past 24 h**			
Mean ± SD (range)	5.0 ± 2.4 (1.0–10.0)	4.0 ± 1.5 (2.0–6.0)	0.37
**Q7 - Level of pain when it is most painful in the past 2 weeks**			
Mean ± SD (range)	7.9 ± 2.6 (2.0–10.0)	6.2 ± 2.6 (2.0–10.0)	0.08
**Q8 - Level of pain when it is least painful in the past 2 weeks**			
Mean ± SD (range)	3.7 ± 3.2 (0.0–9.0)	3.3 ± 2.3 (1.0–7.0)	0.57
**Q9 - Level of pain on average in the past 2 weeks**			
Mean ± SD (range)	5.8 ± 2.4 (2.0–10.0)	5.0 ± 2.4 (2.0–10.0)	0.24
**Q10 - Level of worst non-eye pain in the past 24 h**			
Mean ± SD (range)	3.4 ± 3.8 (0.0–10.0)	2.8 ± 2.7 (0.0–7.0)	0.79
**Q11 - Level of worst non-eye pain in the past 2 weeks**			
Mean ± SD (range)	4.3 ± 4.3 (0.0–10.0)	3.0 ± 3.0 (0.0–8.0)	0.62
**Q12 - Percentage of time you spend thinking about your non-eye pain (%)**			
Mean ± SD (range)	44.3 ± 40.6 (0–100.0)	32.0 ± 40.8 (0–100.0)	0.61
**Q13 - How much your pain has interfered with/affected reading and/or computer use**			
Mean ± SD (range)	6.6 ± 2.5 (0.0–10.0)	6.0 ± 3.0 (2.0–10.0)	0.53
**Q14 - How much your pain has interfered with/affected driving and/or watching TV**			
Mean ± SD (range)	5.1 ± 3.0 (0.0–9.0)	5.1 ± 2.6 (2.0–10.0)	0.63
**Q15 - How much your pain has interfered with/affected general activity (walking, doing house chores)**			
Mean ± SD (range)	4.9 ± 3.2 (0.0–10.0)	4.4 ± 3.6 (0.0–10.0)	0.78
**Q16 - How much your pain has interfered with/affected mood**			
Mean ± SD (range)	7.5 ± 3.1 (0.0–10.0)	4.5 ± 3.1 (0.0–10.0)	0.06
**Q17 - How much your pain has interfered with/affected sleep**			
Mean ± SD (range)	4.3 ± 3.9 (0.0–10.0)	1.5 ± 1.7 (0.0–4.0)	0.18
**Q18 - How much your pain has interfered with/affected enjoying life/relations with other people**			
Mean ± SD (range)	6.9 ± 3.4 (0.0–10.0)	4.1 ± 3.7 (0.0–10.0)	0.10
**Q19 - Percentage of time you spend thinking about your eye pain (%)**			
Mean ± SD (range)	72.3 ± 30.5 (10.0–100.0)	77.1 ± 22.8 (50.0–100.0)	0.83
**Q20 - How much your pain increased when exposed to wind, dry air, heat, air conditioning (%)**			
Mean ± SD (range)	53.9 ± 37.8 (0.0–100.0)	68.5 ± 24.1 (50.0–100.0)	0.40
**Q21 - How much your pain increased when exposed to volatile chemicals (cleaning agents, fumes, cosmetic fragrances) (%)**			
Mean ± SD (range)	28.8 ± 33.4 (0.0–100.0)	41.4 ± 51.7 (0.0–100.0)	0.75
**Q22 - How often your eye pain accompanied by redness (%)**			
Mean ± SD (range)	33.0 ± 37.1 (0.0–100.0)	54.2 ± 37.3 (0.0–100.0)	0.29
**Q23 - How often your eye pain accompanied by burning (%)**			
Mean ± SD (range)	54.3 ± 34.2 (0.0–100.0)	80.0 ± 18.2 (50.0–100.0)	0.10
**Q24 - How often your eye pain accompanied by sensitivity to light (%)**			
Mean ± SD (range)	43.6 ± 39.0 (0.0–100.0)	60.7 ± 44.5 (0.0–100.0)	0.36
**Q25 - How often your eye pain accompanied by tearing (%)**			
Mean ± SD (range)	21.0 ± 25.6 (0.0–100.0)	22.8 ± 30.9 (0.0–80.0)	0.73
**Q26 - How much eye pain relief you have experienced since the last visit (%)**			
Mean ± SD (range)	16.2 ± 23.8 (0.0–70.0)	17.5 ± 28.7 (0.0–60.0)	0.80
**Q27 - How much non-eye pain relief you have experienced since the last visit (%)**			
Mean ± SD (range)	16.6 ± 23.8 (0.0–70.0)	20.0 ± 40.0 (0.0–80.0)	0.89

RS-NCP: Neuropathic corneal pain due to refractive surgery, H-NCP: Neuropathic corneal pain due to herpetic eye disease.
